# Supplementary material for: Variant-specific pathophysiological mechanisms of AFF3 differently influence transcriptome profiles
Source: Genome Med. 2024 May 30;16:72. doi: 10.1186/s13073-024-01339-y (PMC11137988; doi:10.1186/s13073-024-01339-y)
Supplement: Supplementary file 1 — Additional file 1: Figure S1. 3D protein modelling of the human AFF3 degron region bound to SIAH ubiquitin ligase. Figure S2. Sequencing read profiles of the DUP1 individual. Figure S3. Immunostaining of hindbrain neurons and motoneurons in 3dpf zebrafish. Figure S4. AFF3 expression levels in engineered isogenic HEK293T cells. Figure S5. Examples of differential expressed genes (DEGs) loci bound by AFF3. Figure S6. Gene Set Enrichment Analysis for hallmark pathways of DEGs in biallelic loss-of function (LoF/LoF) AFF3 lines and heterozygote loss-of function (Lof/+) AFF3 lines upon comparison with unmutated wildtype lines. [file 13073_2024_1339_MOESM1_ESM.docx]

**Supplementary Figures**


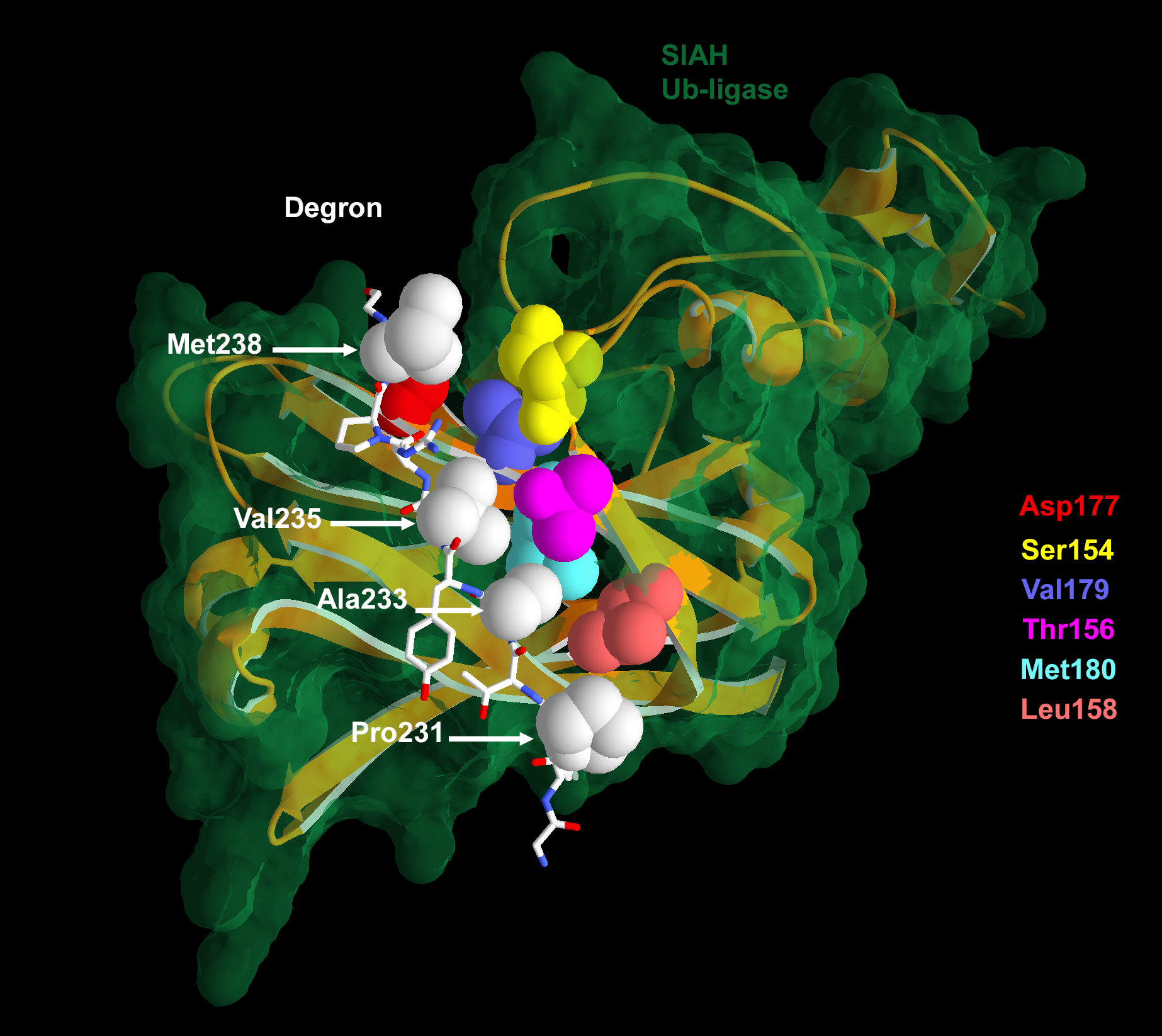
**Figure S1**

**Figure S1. 3D protein modelling of the human AFF3 degron region bound to SIAH ubiquitin ligase.** The AFF3 degron chain is shown as a white stick structure, with residues mutated in KINSSHIP affected individuals highlighted in white space-fill, from top to bottom: Met238, Val235, Ala233, and Pro231. The SIAH ubiquitin ligase is presented as an orange ribbon embedded in its green transparent surface. Amino acids interacting with the degron residues sidechains are clustered in two spatial regions along beta strands and represented spacefilled as follow: Ser154 yellow, Thr156 burgundy Leu158 salmon and Asp177 red, Val179 blue, Met180 cyan.

**Figure S2**

A

**
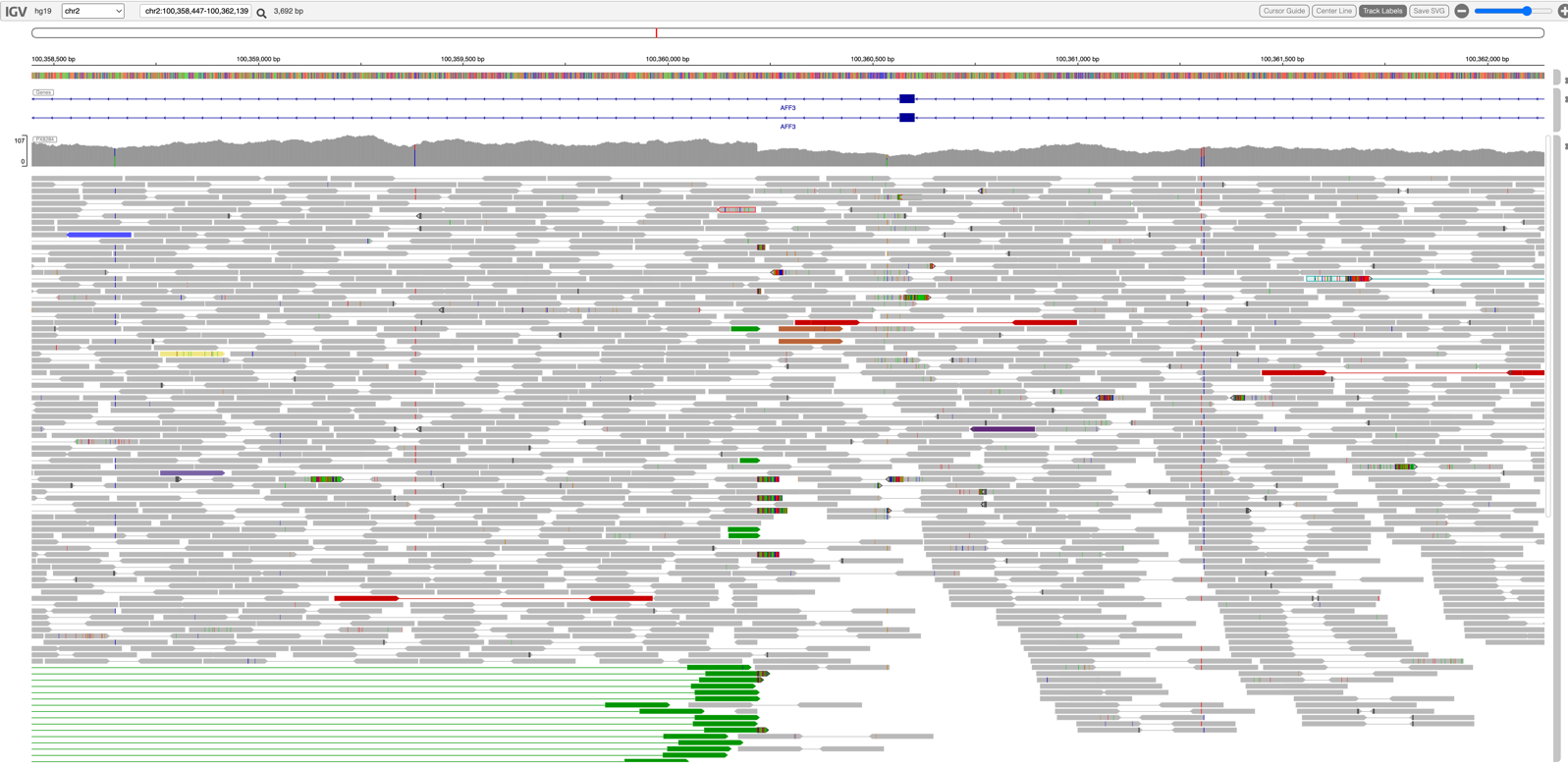
**

B

**
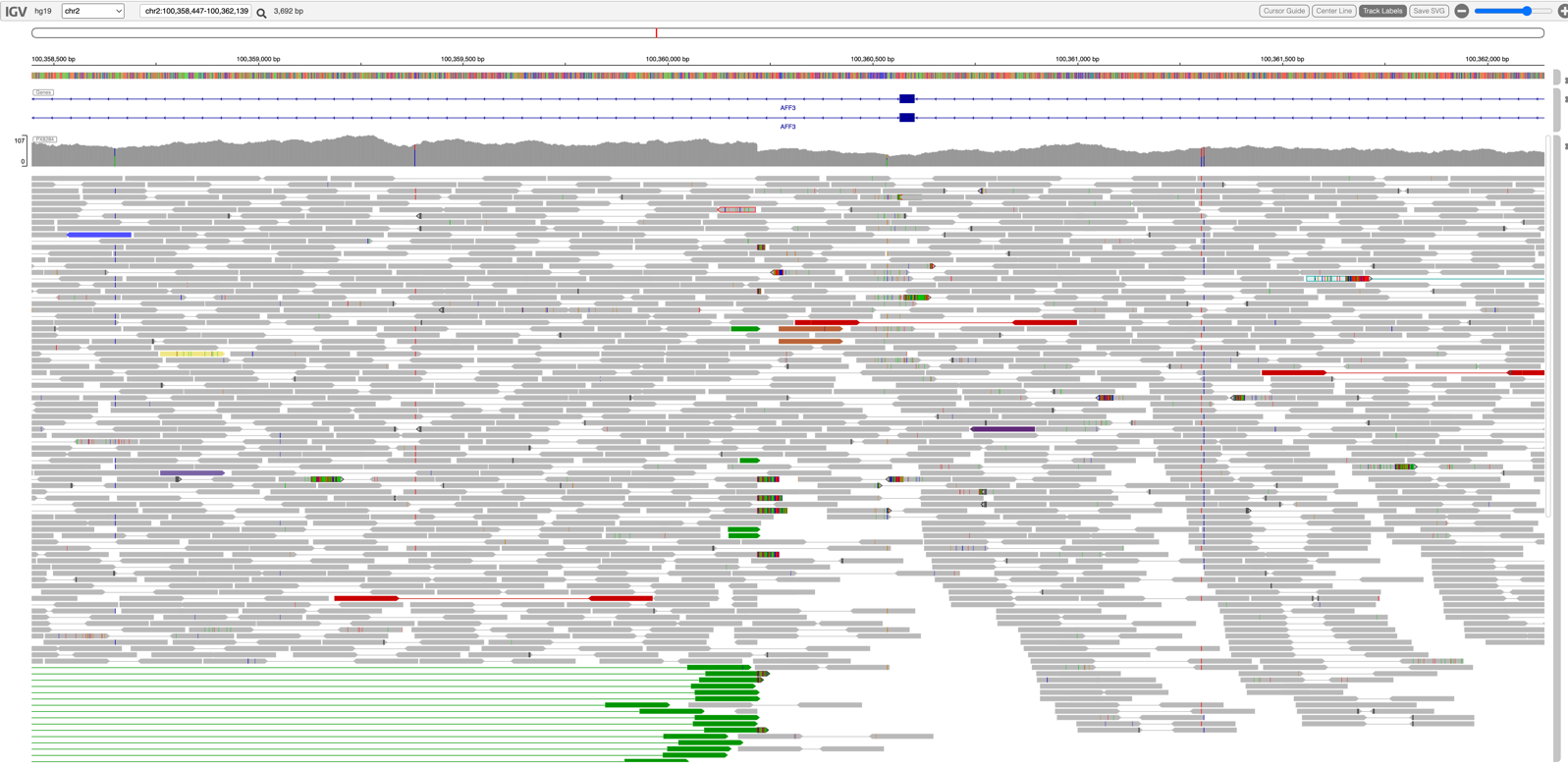
**

**Figure S2. Sequencing read profiles of the DUP1 individual.**

Integrative Genomics Viewer (IGV) sequencing reads profiles of the DUP1 patient at the proximal **(A)** and distal breakpoints **(B)** showing that the duplication that encompasses exon 10 to exon 24 of *AFF3* and exon 1 to 3 of *REV1* is in tandem. Indicative of a tandem duplication, sequencing reads (highlighted in green) at the end of the first duplicated segment are anomalously paired with the reads from the beginning of the second segment.

**Figure S3**


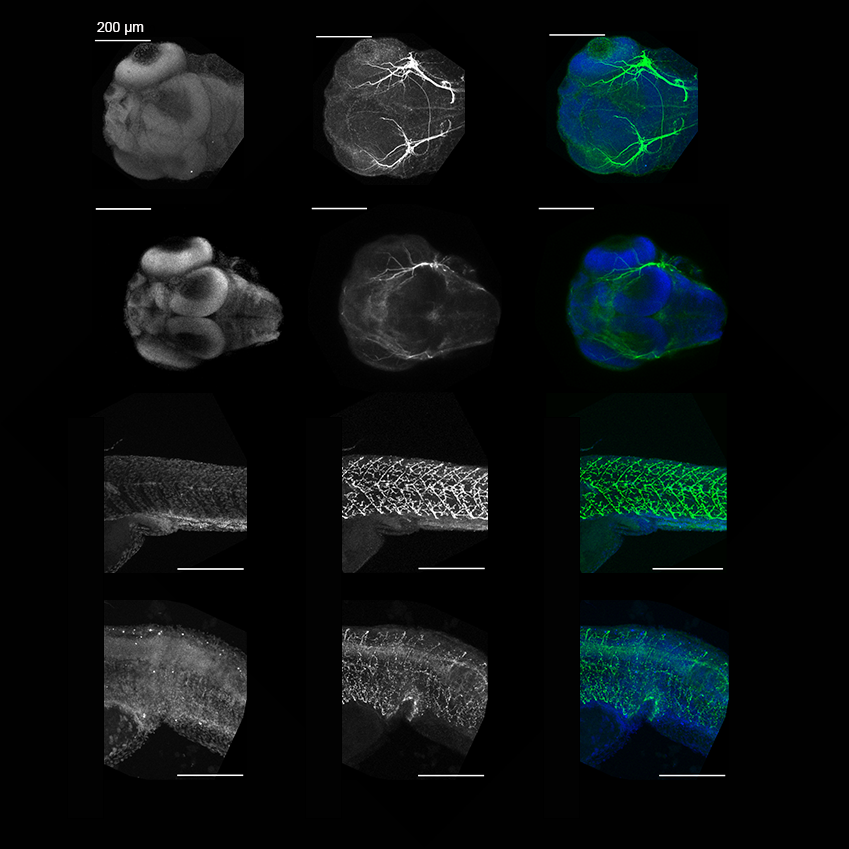
 A

B

**Figure S3. Immunostaining of hindbrain neurons and motoneurons in 3dpf zebrafish.** Maximum projections of confocal images regarding hindbrain neuronal structures **(A)** and motoneurons **(B)** in 3dpf Mock-injected (Mock) and *aff3* KD larvae.

**Figure S4.**

**Figure S4. AFF3 expression levels in engineered isogenic HEK293T cells.**

Dotblot of the VST-normalized (r-log) counts of the *AFF3* gene across all HEK293T engineered samples. We engineered five biallelic LoF HEK293T lines (LoF/LoF) with different combinations of variants (lines No.20 and 98: stop-gain/stop-gain; No.15: stop-gain/20bp deletion; No.4: 4bp deletion/114bp deletion; No.216: 94bp deletion/94bp deletion), one heterozygous LoF stop-gain/+ line (No.1), two homozygous Ala233Thr/Ala233Thr KINSSHIP/KINSSHIP lines (No.54 and 90; DN/DN) and two compound heterozygous KINSSHIP and LoF lines (No.51 and 86: Ala233Thr/stop-gain). The variant nomenclature of the engineered variants is specified in the materials and methods section.

**Figure S5**

**Figure S5. Examples of DEGs** **loci bound by AFF3.**

UCSC genome browser snapshot of the CDK5RAP2, CNNM2, CTNNA3 and DYN*C2I2* loci a bound by AFF3 showing from to top to bottom AFF3 ChIP-seq HEK293T results, UCSC and REFSeq curated gene structure and vertebrate PhyloP conservation scores (left panels). Expression level of the corresponding DEGs in +/+ (blue), LoF/LoF (yellow) and KINSSHIP/KINSSHIP (DN/DN; green) HEK293T engineered lines (right panels).

**
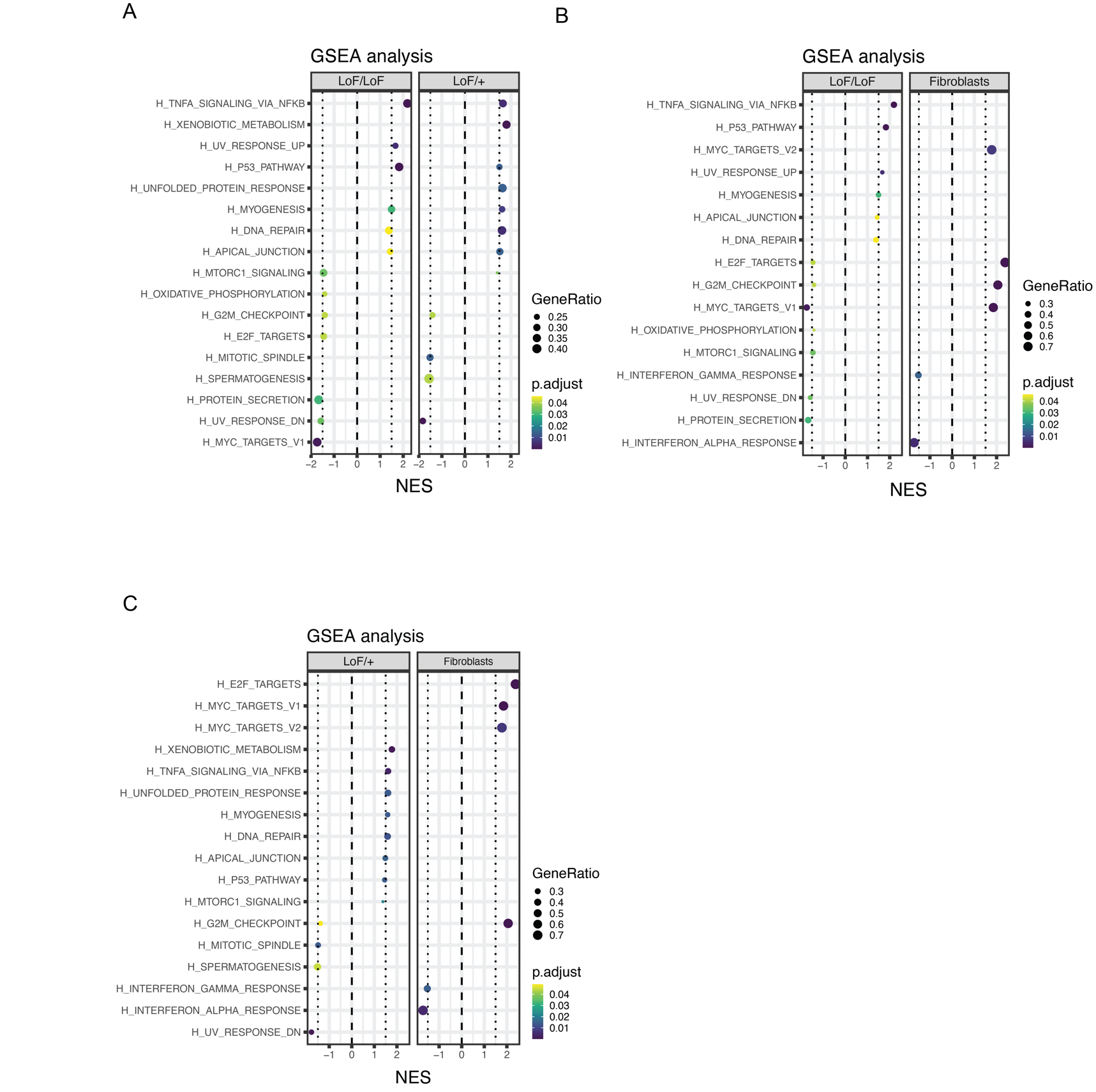
Figure S6**

**Figure S6. Gene Set Enrichment Analysis**

Gene Set Enrichment Analysis (GSEA) for hallmark pathways of DEGs **(A)** in biallelic loss-of function (LoF/LoF) *AFF3* lines (left panel) and heterozygote loss-of function (Lof/+) *AFF3* lines (right panel) upon comparison with unmutated wildtype lines; **(B)** in biallelic loss-of function (LoF/LoF) *AFF3* lines (left panel) and fibroblasts of probands (fibroblasts; right panel) upon comparison with unmutated wildtype lines and fibroblasts of controls, respectively; and **(C)** in heterozygous loss-of function (LoF/+) *AFF3* lines (left panel) and fibroblasts of probands (fibroblasts; right panel) upon comparison with unmutated wildtype lines and fibroblasts of controls, respectively.
