# Supplementary material for: Variant-specific pathophysiological mechanisms of AFF3 differently influence transcriptome profiles
Source: Genome Med. 2024 May 30;16:72. doi: 10.1186/s13073-024-01339-y (PMC11137988; doi:10.1186/s13073-024-01339-y)
Supplement: Supplementary file 2 — Additional file 2. In Table S1 and Table S2 are described the genotypes and phenotypes of KINSSHIP individuals and of other carriers of AFF3 variants, respectively. [file 13073_2024_1339_MOESM2_ESM.docx]

| **Table S1** | | |  |  |  |  |  |  |
| --- | --- | --- | --- | --- | --- | --- | --- | --- |
| **ID patient** | K1 - K19, Voisin et al. AJHG 2021 (for comparison) | K22 | K23 | K24 | K25 | K26, DDD study, Nature 2017, sample DDD4K.02548 | K27, DDD study, Nature 2017, sample DDD4K.00047 | DUP1 |
| **Variant in AFF3**  **Ref ENST00000317233.4** |  | GRCh37:2:100623254:A>G, NM_002285.3:c.713T>C, NP_002276.2:p.(Met238Thr) | GRCh37:2:100623255:T>C,  NM_002285.3:c.712A>G,  NP_002276.2:p.(Met238Val) | GRCh37:2:100623276:G>A, NM_002285.3:c.691C>T, NP_002276.2:p.(Pro231Ser) | GRCh37:2:100623270:C>T  NM_002285.3: c.697G>A,  NP_002276.2: p.(Ala233Thr) | GRCh37:2:100623270:C>T  NM_002285.3: c.697G>A,  NP_002276.2: p.(Ala233Thr) | GRCh37:2:100623270:C>A  NM_002285.3: c.697G>T,  NP_002276.2: p.(Ala233Ser) | chr2:g.100077649_100359928dup (hg19) (NC_000002.11:g.100077649_100359928dup) |
| **GnomAD** | All not reported | Not reported | Not reported | Not reported | Not reported | Not reported | Not reported | Not reported |
| **Prediction tools** |  | SIFT = 0, POLYPHEN = 0.99, CADD = 23.6, REVEL = 0.623 | SIFT = 0, CADD = 20.6, REVEL = 0.56 | SIFT = 0, POLYPHEN = 0.99, CADD = 25.4, REVEL = 0.769 | CADD=27.1, REVEL = 0.608 | CADD=27.1, REVEL = 0.608 |  |  |
| **Status of the variant** | Heterozygote | Heterozygote | Heterozygote | Heterozygote | Heterozygote | Heterozygote | Heterozygote | Heterozygote |
| **Inheritance** | All *de novo* | *de novo* | *de novo* | *de novo* | *de novo* | *de novo* | *de novo* | *de novo* |
| **Year of birth** |  | 2015 | 2005 | 2019 | 2004 |  |  | 2007 |
| **Sex** |  | F | M | F | M | M | M | F |
| **Growth restriction** | Failure to thrive 14/18 (78%) | No | Yes, height 167 cm (-2.2SD), target height (-0.5SD) | No | Weight gain normal, height in -4SD |  |  | Feeding problems |
| **Brain** | Global brain atrophy and or ventriculomegaly 13/15 (87%) | No | No | Hypoplasia of corpus callosum, abnormal cortical gyration | Normal (slightly wider ventricles) |  |  | Mild periventricular leukopathy |
| **Epileptic encephalopathy, epilepsy** | Epileptic encephalopathy 14/18 (78%) | No | No | No | Focal onset epilepsy at 8yo |  |  | Epileptiform activity, spike and wave bifrontal discharge |
| **Facial dysmorphism** | Yes: bulbous nasal tip 10/15 (67%), wide mouth 10/16 (63%), square upper lip, abnormalities of teeth and gums 12/15 (80%) | Long face, broad nasal ridge, low columella | Long palpebral fissures, bulbous nose, wide spaced teeth, full lips | Down slanted palpebral fissures, bilateral epicanthal folds, anteverted nostrils | Epicanthal folds, bushy eyebrows, down slanted palpebral fissures, almond-shaped eyes, anteverted nostrils, tented upper lip, everted lover lip, small mouth, widely spaced teeth, slightly backward rotated ears |  |  |  |
| **Cranial dysmorphism** | Microcephaly 9/18 (50%) | No | HC +1.3SD | Dolichocephaly | HC -2SD |  |  |  |
| **DD/ID** | DD and/or ID 18/18 (100%) | Mild DD | DD and ID | DD and ID | DD | DD | DD | Severe DD (short eye contact, not able to sit) |
| **Social interaction disorder** |  | No | ASD | Yes | Short eye contact |  |  |  |
| **Short attention span** |  | Yes | no | No data | NA |  |  | Yes |
| **Speech impairment** |  | Mild | Mild | Severe | No speech |  |  | No speech |
| **Hypertrichosis** | yes 12/15 (80%) | Mild | No | Yes | Yes |  |  |  |
| **Muscle** | Abnormalities of muscle tone 12/16 (75%) | History of mild hypotonia, improved | History of hypotonia, improved | Low muscle tone | Hypotonia (trunk and head), spasticity in the extremities, contractures. Botox injections as treatment |  |  | Hypotonia (higher lower limb tone), contractures (wrists, hips), poor head control |
| **Skeletal defects** | Mesomelic dysplasia 12/18 (67%); Fibular hypoplasia 12/16 (75%); scoliosis 9/15 (60%) | Sacral dimple | Mild scoliosis | Mesomelic dysplasia, short tibia and fibula and sacral dimple | Mesomelic dysplasia, absent fibula (left), hypoplastic fibula (right), arthrogryposis multiplex congenita, also abnormalities in the upper extremities, scoliosis (operated) and kyphosis |  |  | Severe scoliosis |
| **Finger/Toe anomalies** |  | 4th and 5th toe clinodactyly, wide proximal phalanx of 2nd finger, prominent fingertip pads | Prominent big toes | Camptodactyly | Feet: 4^th^ and 5^th^ toe metatarsal are fused, syndactyly, sandal gap  Hands: pterygium between 1^st^ and 2^nd^ finger, syndactyly |  |  |  |
| **Kidney defects** | Horseshoe or hypoplastic kidney 13/17 (76%) | No | NA | No | Horseshoe kidney |  |  |  |
| **Respiratory system defects** | 8/17 (47%) | No | Obstructive sleep apnea | No | Slow swallowing, prone to aspiration pneumonia, weak cough |  |  |  |
| **Others** | Gastroesophageal reflux disease 6/16 (38%) and other gastrointestinal symptoms 14/17 (82) | Hypoplastic 5th toenails; history of Duane retraction syndrome | Obesity: weight +3.7 SD | Deep plantar creases, external genital hypoplasia | Gastroesophageal reflux, constipation, hypopastic toenails (2-5^th^), strabismus (esotropia), astigmatismus, paraphimosis, G-tube |  |  | Muscle biopsy (EMG) reported normal |

| **Table S2** | | | | | | | | | | | | | | | | | | | | | | | | | |
| --- | --- | --- | --- | --- | --- | --- | --- | --- | --- | --- | --- | --- | --- | --- | --- | --- | --- | --- | --- | --- | --- | --- | --- | --- | --- |
| **ID Patient** | L1 | L2 | L3 | L4 | L5 | L6 | L7  DECIPHER sample 281982 | L8  DECIPHER  Sample 305794 | L9 | L10 | L11 | L12 | L13 | L14 |  | B1 | B2 | B3 | B4  Harripaul, Mol Psy 2018, family PK113 | B5  Harripaul, Mol Psy 2018, family PK113 | B6  Harripaul, Mol Psy 2018, family PK113 | B7 |  | M1 | M2  DDD study, Nature 2017, sample DDD4K.02548 |
| **Family** | Family 1 | Family 1 | Family 1 | Family 1 | Family 2 | Family 3 | Family 4 | Family 5 | Family 6 | Family 6 | Family 7 | Family 8 | Family 8 | Family 8 |  | Family 9 | Family 10 | Family 10 | Family 11 | Family 11 | Family 11 | Family 12 |  | Family 13 | Family 14 |
| **Family member** | Proband | Brother 1 | Brother 2 | Father | Proband | Proband | Proband | Proband | Father | Proband | Proband | Proband | Brother | Mother |  | Proband | Proband | Sister | Affected 1 | Affected 2 | Affected 3 | Proband |  | Proband | Proband |
| **Variant 1**  **Ref ENST00000317233.4** | GRCh37:2:100170944:CGGAGCTGGCG>C; NM_002285.3:c.3378_3387delGGAGCTGGCG, NP_002276.2:p.(Ala1127TrpfsTer134) cDNA.3614_3623delCGCCAGCTCC | GRCh37:2:100170944:CGGAGCTGGCG>C; NM_002285.3:c.3378_3387delGGAGCTGGCG, NP_002276.2:p.(Ala1127TrpfsTer134) cDNA.3614_3623delCGCCAGCTCC | GRCh37:2:100170944:CGGAGCTGGCG>C; NM_002285.3:c.3378_3387delGGAGCTGGCG, NP_002276.2:p.(Ala1127TrpfsTer134) cDNA.3614_3623delCGCCAGCTCC | GRCh37:2:100170944:CGGAGCTGGCG>C; NM_002285.3:c.3378_3387delGGAGCTGGCG, NP_002276.2:p.(Ala1127TrpfsTer134) cDNA.3614_3623delCGCCAGCTCC | 570kb deletion of 2q11.2 (GRCh37,NC_000002.11:g.100456588_101026865del) | GRCh37:2:100199329:A>AAACAAACTGTTGCC, NM_002285.3:c.2710_2723dup, NP_002276.2:p.(Phe908LeufsTer85) | arr[GRCh38] 2q11.2(99644464_100282475)x1 pat(GRCh37_100260927_100898938del) | 744.09 kb deletion, GRCh37:2:99487537_100232437del, NC_000002.11:g.99487537_100232437del) | GRCh37:2:100209897G>GA, NM_002285.3:c.2225dup, NP_002276.2:p.(Tyr743LeufsTer19) | GRCh37:2:100209897G>GA, NM_002285.3:c.2225dup, NP_002276.2:p.(Tyr743LeufsTer19) | GRCh37:2:100623679:GC>G, NM_002285.3:c.417del, NP_002276.2:p.(Gln139HisfsTer82) | GRCh37:2:100368748:GA>G, NM_002285.3:c.936del, NP_002276.2:p.(Pro313HisfsTer60) | GRCh37:2:100368748:GA>G, NM_002285.3:c.936del, NP_002276.2:p.(Pro313HisfsTer60) | GRCh37:2:100368748:GA>G, NM_002285.3:c.936del, NP_002276.2:p.(Pro313HisfsTer60) |  | GRCh37:2:100210540:T>C, NM_002285.3:c.1583A>G, NP_002276.2:p.(Lys528Arg) | GRCh37:2:100210342:G:C, NM_002285.3:c.1781C>G, NP_002276.2:p.(Thr594Ser) | GRCh37:2:100210342:G>C, NM_002285.3:c.1781C>G, NP_002276.2:p.(Thr594Ser) | GRCh37:2:100167973:C>A, NM_002285.3:c.3644G>T, NP_002276.2:p.(Gly1215Val) | GRCh37:2:100167973:C>A, NM_002285.3:c.3644G>T, NP_002276.2:p.(Gly1215Val) | GRCh37:2:100167973:C>A, NM_002285.3:c.3644G>T, NP_002276.2:p.(Gly1215Val) | GRCh37:2: 100181962:C>T, NM_002285.3: c.3106G>A, NP_002276.2: p.(Val1036Ile) |  | GRCh37:2:100199397:C:T, NM_002285.3:c.2656G>A, NP_002276.2:p.(Ala886Thr) | GRCh37:2: 100368751 G>A, NM_002285.3:c.934C>T,  NP_002276.2:p.(Leu312Phe) |
| **Variant 1 in Gnomad** | Not reported | Not reported | Not reported | Not reported | Not reported | Not reported | Not reported | Not reported | Not reported | Not reported | Not reported | Not reported | Not reported | Not reported |  | 3.6E-06 | Not reported | Not reported | Not reported | Not reported | Not reported | 3.2E-05 |  | Not reported | Not reported |
| **Prediction tools** |  |  |  |  |  |  |  |  | SpliceAI = 0.99 (splice loss) | SpliceAI = 0.99 (splice loss) | Splice AI = 0.22 (splice loss) |  |  |  |  | SIFT = 0.45, POLYPHEN = 0.029, CADD = 15.57, REVEL = 0.044 | SIFT = 0.09, POLYPHEN = 0.038, CADD = 18.49, REVEL = 0.172 | SIFT = 0.09, POLYPHEN = 0.038, CADD = 18.49, REVEL = 0.172 | SIFT = 1, POLYPHEN = 0.98, CADD = 26, REVEL = 0.723 | SIFT = 1, POLYPHEN = 0.98, CADD = 26, REVEL = 0.723 | SIFT = 1, POLYPHEN = 0.98, CADD = 26, REVEL = 0.723 | SIFT = 0, POLYPHEN = 0.774, CADD = 27.4, REVEL = 0.298 |  | SIFT = 0.006, POLYPHEN = 0.003, CADD = 16.72, REVEL = 0.044 | SIFT = 0.16, POLYPHEN = 0.785, CADD = 24.6, REVEL = 0.247 |
| **Variant 2**  **Ref ENST00000317233.4** | Does not apply | Does not apply | Does not apply | Does not apply | Does not apply | Does not apply | Does not apply | Does not apply | Does not apply | GRCh37:2:100182009:T>C, NM_002285.3:c.3059A>G, NP_002276.2:p.(Gln1020Arg) | GRCh37:2:100623679:GC>G, NM_002285.3:c.417del, NP_002276.2:p.(Gln139HisfsTer82) | GRCh37:2:100368748:GA>G, NM_002285.3:c.936del, NP_002276.2:p.(Pro313HisfsTer60) | GRCh37:2:100368748:GA>G, NM_002285.3:c.936del, NP_002276.2:p.(Pro313HisfsTer60) | Does not apply |  | GRCh37:2:100210540:T>C, NM_002285.3:c.1583A>G, NP_002276.2:p.(Lys528Arg) | GRCh37:2:100210342:G:C, NM_002285.3:c.1781C>G, NP_002276.2:p.(Thr594Ser) | GRCh37:2:100210342:G>C, NM_002285.3:c.1781C>G, NP_002276.2:p.(Thr594Ser) | GRCh37:2:100167973:C>A, NM_002285.3:c.3644G>T, NP_002276.2:p.(Gly1215Val) | GRCh37:2:100167973:C>A, NM_002285.3:c.3644G>T, NP_002276.2:p.(Gly1215Val) | GRCh37:2:100167973:C>A, NM_002285.3:c.3644G>T, NP_002276.2:p.(Gly1215Val) | GRCh37:2:100170775:C>T, NM_002285.3:c.3557G>A, NP_002276.2:p.(Arg1186Gln) |  | Does not apply | Does not apply |
| **Variant 2 in Gnomad** |  |  |  |  |  |  |  |  |  | Not reported | Not reported | Not reported | Not reported |  |  | 3.6E-06 | Not reported | Not reported | Not reported | Not reported | Not reported | 5.8E-04* |  |  |  |
| **Prediction tools** |  |  |  |  |  |  |  |  |  | SIFT = 0.35, Polyphen =0.08, CADD = 19.04 , REVEL = 0.055 | Splice AI = 0.22 (splice loss) |  |  |  |  | SIFT = 0.45, POLYPHEN = 0.029, CADD = 15.57, REVEL = 0.044 | SIFT = 0.09, POLYPHEN = 0.038, CADD = 18.49, REVEL = 0.172 | SIFT = 0.09, POLYPHEN = 0.038, CADD = 18.49, REVEL = 0.172 | SIFT = 1, POLYPHEN = 0.98, CADD = 26, REVEL = 0.723 | SIFT = 1, POLYPHEN = 0.98, CADD = 26, REVEL = 0.723 | SIFT = 1, POLYPHEN = 0.98, CADD = 26, REVEL = 0.723 | SIFT = 0.2, POLYPHEN = 0.04, CADD = 24.26, REVEL = 0.150 |  |  |  |
| **Hetero/Homozygote** | Heterozygote LoF/+ | Heterozygote LoF/+ | Heterozygote LoF/+ | Heterozygote LoF/+ | Heterozygote LoF/+ | Heterozygote LoF/+ | Heterozygote LoF/+ | Heterozygote LoF/+ | Heterozygote LoF/+ | Compound heterozygote LoF/missense | Homozygote LoF/LoF | Homozygote LoF/LoF | Homozygote LoF/LoF | Heterozygote LoF/+ |  | Homozygote missense/missense | Homozygote missense/missense | Homozygote missense/missense | Homozygote missense/missense | Homozygote missense/missense | Homozygote missense/missense | Compound heterozygote missense/missense |  | Heterozygote missense/+ | Heterozygote missense/+ |
| **Inheritance** | AD, haploinsufficiency | AD, haploinsufficiency | AD, haploinsufficiency | AD, haploinsufficiency | AD, **of note, parental testing not completed** | AD, haploinsufficiency | Paternally inherited, AD, haploinsufficiency | AD, haploinsufficiency | AD, haploinsufficiency | AR (Semi-dominant ?) | AR | AR (Semi-dominant ?) | AR (Semi-dominant ?) | AD, haploinsufficiency |  | AR | AR | AR | AR | AR | AR | AR |  | AD,  *de novo* | AD,  *de novo* |
| **Year of birth** | 2018 | 2014 | 2019 | 1993 | 2021, d. 2m of age |  | 1999 |  |  |  | 2023 | 1976 | 1984 | 1958 |  | 2004 | 2012 | 2012 |  |  |  | 2020 |  | 2021 |  |
| **Sex** | M | M | M | M | M | F | F | M | M | M | M | F | M | F |  | F | F | F |  |  |  | M |  | M | F |
| **IUGR** | No |  | No |  | No | Yes | No | Yes | No data | No |  | Yes | Yes | No data |  | Yes, and oligohydramnios | No | No |  |  |  |  |  | No |  |
| **Plagiocephaly** | No | Yes | Yes |  | No |  | No data |  | No data | No |  | No | No data | No data |  | No | No | No |  |  |  | Yes |  | No |  |
| **Ventriculomegaly** | Ventricular prominence, but not ventriculomegaly |  | No |  | Marked enlargement of the subarachnoid spaces with 7 mm depth CSF isointense subdural collection overlying the right cerebral convexity, and trace left frontal subdural collection |  | No |  | No data | No |  | No data | No data | No data |  | No | No | No |  |  |  | No |  | No |  |
| **Epileptic encephalopathy, seizure** | No |  | No |  | Not at time of death |  | No |  | No | No | Central sleep apnea | No | No | No |  | No | Abnormal sleep EEG: spike-waves in left occipital region | Abnormal sleep EEG: focal paroxysmal abnormalities; three episodes of absence seizures |  |  |  | Yes |  | No |  |
| **Abnormal corpus callosum** | No | Yes, abnormally thin | Yes, abnormally thin |  | Brain MRI- marked diffuse cerebral and cerebellar parenchymal volume loss or diminished brain growth | Agenesis of corpus callosum | No data |  | No data | No |  | No data | No data | No data |  | No | No | No |  |  |  | Yes |  | No MRI |  |
| **Abnormal cortical gyration** | No |  | No |  | Abnormal parenchyma per above |  | No data. |  | No data | No |  | No data | No data | No data |  | No | No | No |  |  |  | No |  | No MRI |  |
| **Myelination** | Normal |  | Delayed |  | Not mentioned (only 2m at time of death) |  | No data |  | No data | No |  | No data | No data | No data |  | No | Normal | Normal |  |  |  |  |  | No MRI |  |
| **Facial dysmorphisms** | Low set posteriorly rotated ears, widely spaced eyes |  | Widely spaced eyes |  | None reported | Ocular hypertelorism, broad nasal root, short and upturned nose with depressed nasal bridge, anteverted and hypoplastic nostrils, relatively large and tented mouth with exposed upper gums | No data |  | No data | Diastema, thin upper lip, mild deep-set eyes | Hypertelorism, microretrognathia | upslant, slight hypertelorism, upturned nostrils, microretrognathia, simple ears | No data | No data |  | Synophrys, long eyelashes | No | No |  |  |  | No |  | No |  |
| **Cranial dysmorphisms** | No |  | No |  |  | Dysmorphic craniofacial features and mild micrognathia, microcephaly | No |  | No | Turricephaly | Deep-set ears and downslanting palpebral fissures | No | No data | No data |  | No | No | No |  |  |  | No |  | NO |  |
| **Anal atresia** | No |  | No |  | Imperforated anus |  | No |  | No | No |  | No | No data | No data |  | No | No | No |  |  |  | No |  | NO |  |
| **DD** | Yes, global DD | Yes, global DD | Yes, global DD | Yes, mild cognitive delays | N/a, too young to assess | Yes | Yes, global DD |  | Mild ID | Mild ID | N/a, too young to assess | DD, Severe ID | Mild-moderate intellectual disability | Mild intellectual disability |  | No | DD | DD | ID | ID | ID | DD |  | DD | DD |
| **Autism** | Yes |  | No |  | N/a, too young to assess |  | No |  | No | No |  | No | No data | No data |  | No | No, but ADHD | No, but ADHD |  |  |  |  |  | Possibly | Possibly |
| **Social interaction disorder** | Behavioral therapy is ongoing |  | yes |  | N/a, too young to assess |  | No |  | no data | Yes, in young age |  | Prominent social anxiety | Prominent social anxiety | No data |  | No | No | No |  |  |  | No interaction |  | No data | No data |
| **Short attention span** | Yes |  | No |  | N/a, too young to assess |  | No |  | Yes | Yes, low long-term memory |  | No, corresponding to level of intellectual disability | No data | No data |  | No | Yes | Yes |  |  |  | No attention |  | No data | No data |
| **Speech impairment** | Yes | Yes | Yes | Yes, speech delays and stuttered | N/a, too young to assess | Receptive expressive language disorder | No |  | Yes, severe | Yes, severe | N/a, too young to assess | Yes, severe | Yes | slight dysarthria, simple vocabulary |  | No | Mild, dyslalia | Moderate, poor language skills and dyslalia |  |  |  | No speech |  | Yes, mild | Yes, mild |
| **Hypodontia** | Not assessed |  | No |  | N/a, too young to assess |  | N.D. |  | No | Yes | N/a, too young to assess | No | No data | No data |  | No data | No | No |  |  |  |  |  | No | No |
| **Widely spaced teeth** | Yes |  | No |  | N/a, too young to assess | Yes | N.D. |  | No | Yes |  | No | No data | No data |  | No data  *has been treated with braces for unknown reason | No | No |  |  |  | No |  | No | No |
| **Hypertrichosis** | No |  | No |  | Not noted |  | No |  | No | Yes |  | No | No data | No data |  | Yes, normal androgens | No | No |  |  |  | No |  | No | No |
| **Muscle disorders** | No |  | hyperreflexia and hypotonia |  |  |  | No |  | No | Yes, mild motor hyperkinesia |  | No | No data | No data |  | No | No | No |  |  |  | No |  | No | No |
| **Low muscle tone** | Yes | Yes | Yes |  | Mildly hypotonic | Hypotonia | No | Neonatal hypotonia | No data | No |  | Hypotonia after birth and feeding problems with weak suction during drinking | No data | No data |  | No | No | No |  |  |  | No |  | No | No |
| **Skeletal defects** | Not known |  | No |  | Not noted but skeletal survey could not be completed due to respiratory status | Short stature, brachydactyly, minor exterior skeletal anomalies, short neck | No |  | Not known | Winged shoulder blades |  | Slight hyperlaxity of the metacarpophalangeal joints, slightly long great toes | right clubfoot, right hip dysplasia | No data |  | Bilateral dysmelia. Double tibias. Severe contractures of knees and ankles. Bilateral Pes equinovarus. Asymmetric preaxial polydactyly; 7 toes right side (with 5 seemingly normal toes and two preaxial toes). 8 toes left side (with 2x4 toes where the first toe is missing), the individual toes seem normal with normal nails. Multiple anomalies of costae bilaterally. Femur amputated bilaterally due to lower leg malformations. | No | No |  |  |  | No |  | No | No |
| **Clinodactyly** | No |  | No |  |  | Yes | No |  | No data | No |  | fifth digits | No data | No data |  | No | No | No |  |  |  | No |  | No | No |
| **Heart defects** | No |  | No |  | Tetralogy of Fallot, absent pulmonary valve | Patent foramen ovale, Patent ductus arteriosus | Hypertension |  | No | No |  | No | No data | No data |  | secundum atrial septal defect | Yes, interatrial defect | No |  |  |  | No |  | No | No |
| **Kidney defects** | Yes, prenatal hydronephrosis |  | No |  | Renal US normal, but two adrenal/para-adrenal lesions of unknown etiology | No | No data |  | No | No |  | No | No data | right renal agenesis |  | Horseshoe kidney | No | No |  |  |  | No |  | No data | No data |
| **Respiratory System defects** | No |  | No |  | Yes- respiratory distress, pneumothorax, tracheomalacia | No | No | Abnormal morphology of pulmonary valve | No data | No |  | Recurrent upper respiratory tract infections before puberty | Recurrent upper respiratory infections before puberty, Recurrent pneumonia before puberty | No data |  | Stenosis right upper lobe bronchus, chronic obstructive pulmonary disease | No | No |  |  |  | No |  | No | No |
| **Other symptoms** | Ocular albinism, OCA2 compound heterozygous variants |  |  |  |  | Bilateral sensorineural hearing loss, cystic hygroma, transverse palmar crease, bilateral nasolacrimal duct obstruction, refractive amblyopia, dysphagia, omphalocel, diaphragmatic anomalies | Severe obesity (familial), hypercholesterolemia in childhood |  |  | Broad and long great toes | Cleft palate, clubfoot | Strabismus, rosacea, over-bite and narrow arch of denture, prominent upper incisors, walking at 4 years. |  | Polymyalgia rheumatica |  | Hypoplastic vagina and uterus. Normal ovaries. | Clumsiness, hypermetropia, astigmatism | Clumsiness, dysmetria; dyscalculia; congenital nystagmus, hypermetropia; hyperphagia, brain MRI: tortuosity of the optic nerves |  |  |  | Hypersalivation, Very sick child |  | none | none |
| **NOTES** |  | SNP microarray and Fragile X testing normal | Epilepsy, but not epileptic encephalopathy |  | Other CMA findings- 432 kb duplication of 22q11.21 (hg19, 21,033,397_21,465,659) and ROH in 8.9% of genome |  |  |  | 2 brothers with intellectual disability (nt tested) |  | Central sleep apnea |  | Not able to live without support of mother |  |  | Cerebral MRI normal, but poor quality due to braces, Dark pigmented skin patch right side of chest/mammae (15 cm long, 6 cm wide) | Normal | Normal |  |  |  |  |  |  |  |

Footnotes: *We identified the p.(Arg1186Gln) variant in 945 genotyped alleles out of a total of 1,614,170 alleles investigated or a frequency of 5.85E-04 (similar to the frequency found by GnomAD 4.0) with two homozygous individuals marked as unaffected but not thoroughly phenotyped. Of note this allele is associated with a significant decrease in *AFF3* expression in fibroblasts (see text for details).
